# Effects of Hand Orientation on Motor Imagery - Event Related Potentials Suggest Kinesthetic Motor Imagery to Solve the Hand Laterality Judgment Task

**DOI:** 10.1371/journal.pone.0076515

**Published:** 2013-09-27

**Authors:** Marijtje L. A. Jongsma, Ruud G. J. Meulenbroek, Judith Okely, C. Marjolein Baas, Rob H. J. van der Lubbe, Bert Steenbergen

**Affiliations:** 1 Behavioral Science Institute, Radboud University Nijmegen, Nijmegen, The Netherlands; 2 Cognitive Psychology & Ergonomics, University of Twente, Enschede, The Netherlands; 3 Donders Institute for Brain, Cognition, &Behavior, Donders Centre for Cognition, Radboud University Nijmegen, Nijmegen, The Netherlands; 4 Department of Cognitive Psychology, University of Finance and Management, Warsaw, Poland; University of Reading, United Kingdom

## Abstract

Motor imagery (MI) refers to the process of imagining the execution of a specific motor action without actually producing an overt movement. Two forms of MI have been distinguished: visual MI and kinesthetic MI. To distinguish between these forms of MI we employed an event related potential (ERP) study to measure interference effects induced by hand orientation manipulations in a hand laterality judgement task. We hypothesized that this manipulation should only affect kinesthetic MI but not visual MI. The ERPs elicited by rotated hand stimuli contained the classic rotation related negativity (RRN) with respect to palm view stimuli. We observed that laterally rotated stimuli led to a more marked RRN than medially rotated stimuli. This RRN effect was observed when participants had their hands positioned in either a straight (control) or an inward rotated posture, but not when their hands were positioned in an outward rotated posture. Posture effects on the ERP-RRN have not previously been studied. Apparently, a congruent hand posture (hands positioned in an outward rotated fashion) facilitates the judgement of the otherwise more demanding laterally rotated hand stimuli. These ERP findings support a kinesthetic interpretation of MI involved in solving the hand laterality judgement task. The RRN may be used as a non-invasive marker for kinesthetic MI and seems useful in revealing the covert behavior of MI in e.g. rehabilitation programs.

## Introduction

Motor imagery (MI) refers to a cognitive process during which the representation of a specific motor action is internally simulated without producing an overt body movement [1-4]. At a neurophysiological level, MI is understood as a cognitive process that engages a variety of supraspinal structures, without descending activation of spinal motor neurons [5]. The study of MI has great relevance for the development of physical rehabilitation treatments [6], motor skill learning, and the development of brain-computer interfaces [7]. It is evident that MI is a central topic in many areas of research. However, the underlying mechanisms of MI are still contested [8,9].

Interest in MI has led to the development of many experimental paradigms that attempt to capture its nature. One such paradigm is the hand laterality judgment (HLJ) task [10]. In the HLJ task, participants are presented with images of hands in different orientations and asked to discriminate between right and left hands. If hand drawings would be processed like any other visual object, reaction times (RTs) should increase only with rotation angle. Such a finding would suggest a pure mental rotation strategy to solve the task [9]. Instead, Parsons [10] found that participants took longer to correctly judge hand laterality when they were presented with laterally compared to medially rotated pictures of hands. Since then, this finding has been repeatedly replicated [10-16]. This consistent observation was taken as evidence that participants internally simulate a movement of the hand - thus, engage in MI - to solve the task. Further evidence in support of MI includes equivalent actual and imagined execution times, autonomic nervous system changes and even improved performance for imagined actions [17,18]. Further support that MI originates from covert simulations of movements stems from neuroimaging studies. Using positron emission tomography (PET scan), Roland et al. [19] demonstrated that MI activates areas related to motor behavior including activation of the primary motor cortex, the prefrontal cortex, and the basal ganglia. More recently, functional magnetic resonance imaging (fMRI) studies have also shown that vividly imagining motor actions activates a widespread network in the brain that shows substantial overlap with the areas involved in both motor execution and motor observation [20]. Pascual-Leone [21] extended this line of research by demonstrating that disrupting activity in the primary motor cortex through transcranial magnetic stimulation (TMS) can increase the time needed to perform MI [5]. This implies that involvement of the primary motor cortex is necessary in MI.

To appreciate the cognitive processes involved in MI, one needs to recognize the division between two perspectives on MI [22]. The first is a 1^st^ person body-centered perspective that can be labeled as kinesthetic motor imagery. The second a 3^rd^ person external perspective based on knowledge of what a movement or posture should look like when watching it from the outside [17]. Even though a 3^rd^ person MI seems more visual in nature it proves also to be affected by biomechanical constraints of the imagined movement based on our general knowledge of human movement [5,8,9]. A means to distinguish between 1^st^ person - or kinesthetic MI - and 3^rd^ person MI, is to manipulate the person’s bodily orientation while he orshe performs the MI task. This manipulation should alter kinesthetic 1^st^ person MI whereas it should not affect 3^rd^ person MI.

In a HLJ task by Funk et al. [12],, children (5-6 years old) and adults had to hold their hands in either a regular or default palm down orientation, or in an awkward, inverted palm up orientation. In the default hand orientation (palm down) they observed faster RTs for back view stimuli than for palm view stimuli. This effect was diminished in the inverted position (palm up), i.e. processing of back view stimuli was prolonged when the hand orientation of the participants was inverted. This finding indicates that the time taken for mental rotation of visually presented hands depends on the momentary orientation of the person’s own hand. In addition, the RT results suggested that young children’s MI is more strongly guided by 1st person kinematic processes than adults’ MI [12].

Apart from activation of cortical areas involved in motor execution, it has been proposed that MI must also rely on representations of one’s own body position, suggesting that instantaneous sensorimotor integration plays a role in the capacity to simulate movements thus supporting a kinesthetic view of MI [21,23]. To determine involvement of specifically motor or visual cortex during MI, many groups have started to incorporate neuroimaging methods aimed at defining the structural correlates of MI and the circumstances in which there is strong overlap between action, perception, and MI. Within their functional MRI study, De Lange et al. [22], used a modified version of Parsons’ HLJ task to this end. They found that in addition to biochemical constraints of the presented hand orientation, reaction times were also affected by the participants’ own hand orientations during the task (forearms were rotated 90% inward or placed straight in front). This effect was accompanied by an increased BOLD response in the intraparietal sulcus. They concluded that MI includes the generation of a motor plan and the construction of this plan is dependent on our current anatomical position [22]. Thus, fMRI research that links MI to activation of specific cortical areas adds to behavioral measures such as reaction times. Unlike fMRI, electro-encephalographic (EEG) techniques are known to have adequate superior temporal resolution to capture MI processes in relation to single motor acts, as performed in the HLJ task, preceding judgement RTs [8,24]. We therefore decided to apply EEG to study the effects of posture variations on MI.

Event-related potentials (ERPs) are time-locked voltage fluctuations in the EEG resulting from sensory, cognitive, or motor-evoked neural activity [25]. The aim of the current study was to investigate the effects of hand orientation on MI using ERPs extracted from the ongoing electroencephalogram (EEG) during an adapted version of the HLJ task in which hand orientation was either congruent or incongruent with presented hand stimuli. This is, to our knowledge, the first ERP study to employ such an approach, allowing a more refined temporal understanding of the involved cognitive processes in such a task. In addition, this non-invasive approach to study subtle and transient changes in neurophysiological activity of mainly superficial cortical areas (i.e. the motor cortices) can be easily applied, even during actual motor behavior, in small children and/or patient groups. Observed interference effects induced by hand orientation manipulations would thus support the view that knowledge about action does indeed depend on a 1^st^ person perspective [26]. Several studies have employed ERP measures of MI in relation to the HLJ task. Thayer and Johnson [14] found that ERP’s recorded during MI displayed similar amplitude modulations as those characteristic of mental rotation. This ERP component, referred to as the rotation related negativity (RRN) is superimposed on the P300. Thus, the P300 becomes less positive as the mentally rotated angle increases. A number of variables have been found to influence RRN amplitude during the HLJ task [14,16]. First, the RRN is more marked for palm view compared to back view pictures of hands. Second, RRN is more marked for laterally rotated than medially rotated pictures of hands. Third, RRN is more marked for left than right hands.

In relation to these findings, the main research question of the present study is whether manipulations of hand postures affect the RRN amplitudes. Such a finding would further support a 1^st^ person, kinesthetic MI to be employed in solving the HLJ task. Based on the literature it is hypothesised that RRNs are less marked for congruent hand postures with pictures of hand stimuli and more marked with incongruent hand postures with pictures of hand stimuli. The current study employed a modified version of the HLJ task. Stimuli were selected from a stimulus set previously used by our research group and reported to elicit the MI specific rotation effect on palm view stimuli [16,33]. Because previous studies have repeatedly demonstrated RT effects, we employed a delayed response version of this task to avoid EEG artefacts. More specifically, we focused on the ERPs recorded from participants during three separate conditions. In each condition hands were in a different position during execution of the hand laterality judgement task. In a control condition hands were kept at a straight position; in one condition the hands were positioned 45° inward; in one condition hands were positioned 45° outward. We recorded the RRN superimposed on the P300 component of the visual ERPs elicited in the HLJ task and determined concurrent efficiency scores in a delayed response task.

## Methods

### Participants

The participant group consisted of 10 healthy right handed volunteers (1 male; 9 female) aged 22.1 ± 2.36 years. Participants were asked to refrain from drinking coffee and smoking cigarettes on the day of the experiment.

### Ethics Statement

Approval of the local ethics committee was obtained – ECG (ethische comissie gedragswetenschappen / ethical committee behavioral sciences, Radboud University Nijmegen, the Netherlands) nr. ECG30062011. All participants signed a written consent form.

### Procedure

**Figure 1a:**
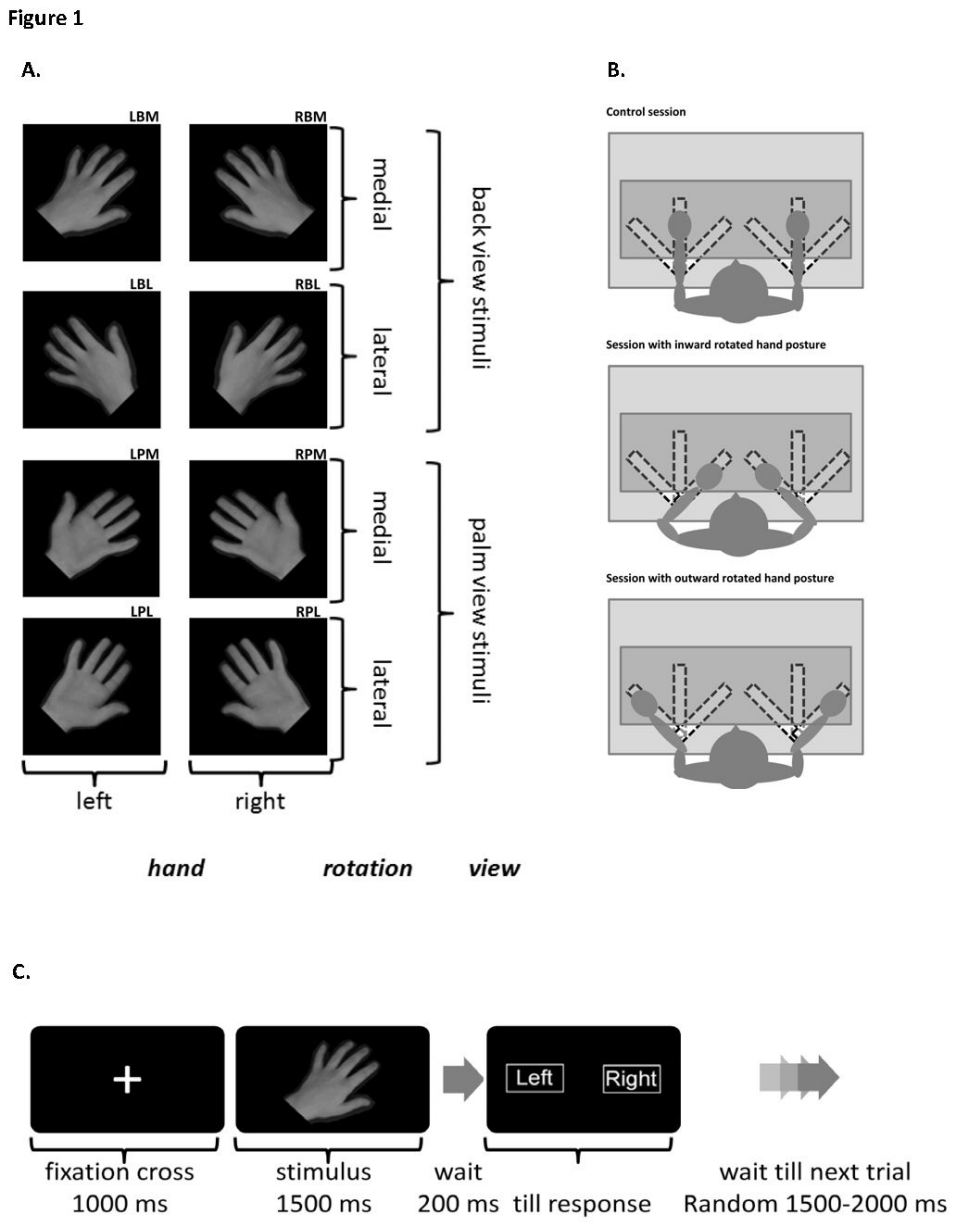
Figure 1a shows the 8 different hand stimuli that were randomly presented during the experiment. Coding of the 8 different stimuli: RBM right hand, back view, medially rotated; RBL, right hand, back view, laterally rotated; LBM left hand, back view, medially rotated; LBL, left hand, back view, laterally rotated; RPM right hand, palm view, medially rotated; RPL, right hand, palm view, laterally rotated; LPM left hand, palm view, medially rotated; LPL, left hand, palm view, laterally rotated; Figure 1b shows a graphic description of the three different hand posture conditions. Figure 1c depicts the presentation order within a trial.

Participants were seated at a desk facing a computer screen with their hands placed on the desk in front of them. Visual stimuli were presented on the screen. Based on a previous experiment of our research group, stimuli consisted of photos of either the palm- or back-view of a right or left hand [32]. The hand pictures were rotated laterally or dorsally by 45 degrees (see Figure 1a). In total, 8 different hand stimuli were used in the experiment. Hand pictures were presented against a plain black background on a computer monitor. Participants performed a HLJ task, i.e., they had to judge whether the presented stimulus depicted a left or right hand. To avoid motor artefacts, the decision had to be indicated after a fixed interval 1700 ms interval (delayed response). Three separate conditions were recorded (see below). The order of the three conditions was counterbalanced over participants via a Latin square.

- In a control condition, participants were asked to keep their hands positions in a straight, 0° rotation position.
- In a condition with an inward rotated hand orientation, participants were asked to position their hands in a 45° inward rotated position.
- In a condition with an outward rotated hand orientation, participants were asked to position their hands in a 45° outward rotated position.

Participants were instructed to assume one of the three positions described above before the start of the recording condition. The hands were placed in the correct position by means of clearly visible markings on the desk (see also Figure 1b). In addition, participant’s hands were covered during the judgement task to prevent the use of visual matching strategies. Participants were instructed to indicate whether the hand stimulus on the screen was a right hand, in which case a response with the right hand was required, or a left hand, in which case a response with the left hand was required. Responses were recorded from two buttons (one below each hand). Participants were instructed to respond as quickly and accurately as possible when the response screen appeared, showing the words “left” and “right” on the corresponding sites of the screen. Every recording session started with four practice trials. The average duration of each session was 30 minutes. For each condition, participants were presented with 80 trials; each trial consisted of a fixation cross (duration 1500 ms) followed by a hand stimulus (duration 1500 ms) followed 200 ms later by a response screen. This delayed response task was used in order to avoid motor artefacts during ERP generation but still allowed the experimenter to exclude incorrectly judged trials from the ERP average. After the response, a random 1500-2000 ms interval occurred before the start of the next trial (see also Figure 1c).

### EEG recordings

EEG signals were recorded with a 32-channel actiCap (MedCaT B.V. Netherlands) and subsequently amplified by a 32-channel BrainAmp EEG amplifier with electrode placement according to the International 10-20 system [27,28]. A ground electrode was placed over AFz and a reference over the left mastoid bone. The EEG signal was offline re-referenced to linked mastoids and stored on disk for offline analyses. Vertical and horizontal eye movements were recorded by two additional bipolar channels placed above and below the right eye and on the outer canthi of each eye. Electrode impedance was kept below 5 kΩ. The signal was digitized at 1000 Hz and filtered online between 0.016 Hz (i.e., 10s time-constant) and 250 Hz.

### Data analysis

**Figure 2:**
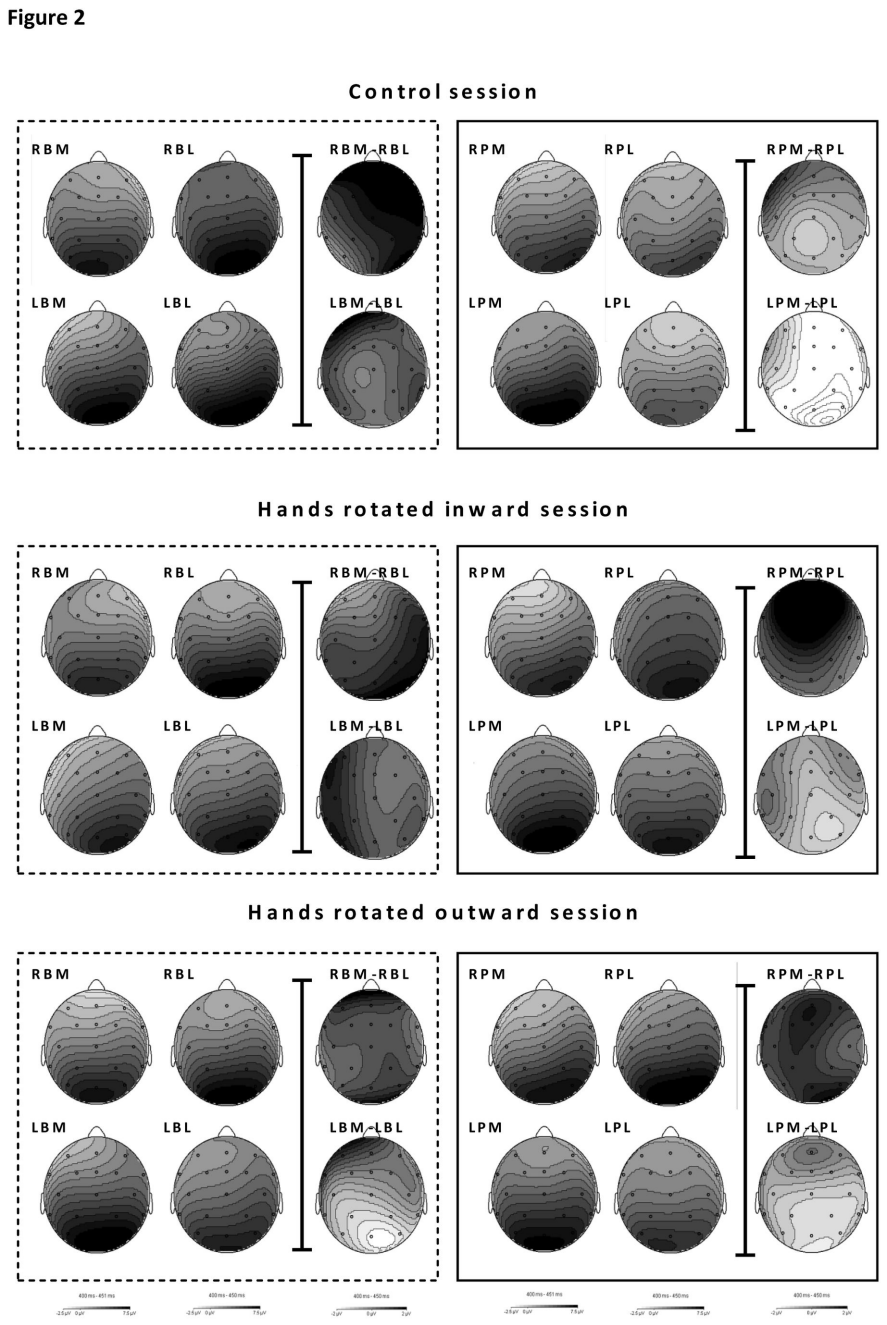
Figure 2 shows the scalp topographies of averaged values in the RRN 400-450 window for the control condition, hands rotated inwards condition and hands rotated outward conditions from top to bottom. Scalp topographies are depicted for 8 stimulus types (RBM, RBL, LBM, LBL, RPM, RPL, LPM, LPL) as well as the scalp topographies of the rotation effects (RBM-RBL; LBM-LBL; RPM-RPL; LPM-LPL). All greyscales range from -2.5 to 7.5 µV for the 8 stimulus types and from -2 tot 2 µV for the rotation effects.

A delayed response task was presented. First, erroneous trials were removed from the data set. EEGs were offline segmented with advanced Boolean expressions that excluded incorrect responses. Segments started 500 ms before stimulus presentation until 1200 ms after stimulus presentation. EEGs were corrected for EOG artefacts by means of the Gratton and Coles algorithm [29] using high- and low-pass filters of 0.5 and 30 Hz (48 dB), respectively. A baseline correction procedure (-500 ms-0 ms) was applied. Resulting segments were averaged per stimulus type to obtain the corresponding ERP. Visual inspection of the grand averaged ERPs revealed that the P300 and superimposed RRN were maximal over Pz, which accords with previous research [16]. The RRN was defined as the average value (µV) within a time-window from 400-450 ms at Pz [16]. See figure 2 for scalp topographies in the 400-450 ms latency window. Inverse efficiency scores (IES) were determined as the RT divided by the proportion of correct responses, expressed in ms [42]. This method is considered to be especially useful in tasks with low (<10%) error rates [42]. Indeed, error rates of the current experiment remained below <6%). IES scores and RRNs were analyzed per posture condition with hand (right hand or left hand), view (palm view or back view), and rotation (45° lateral or 45° medial) as within subject variables. Whenever interaction effects were observed appropriate post-hoc tests, either per view or per hand, were performed with Bonferroni correction.

## Results

### Hands in control position

**Figure 3:**
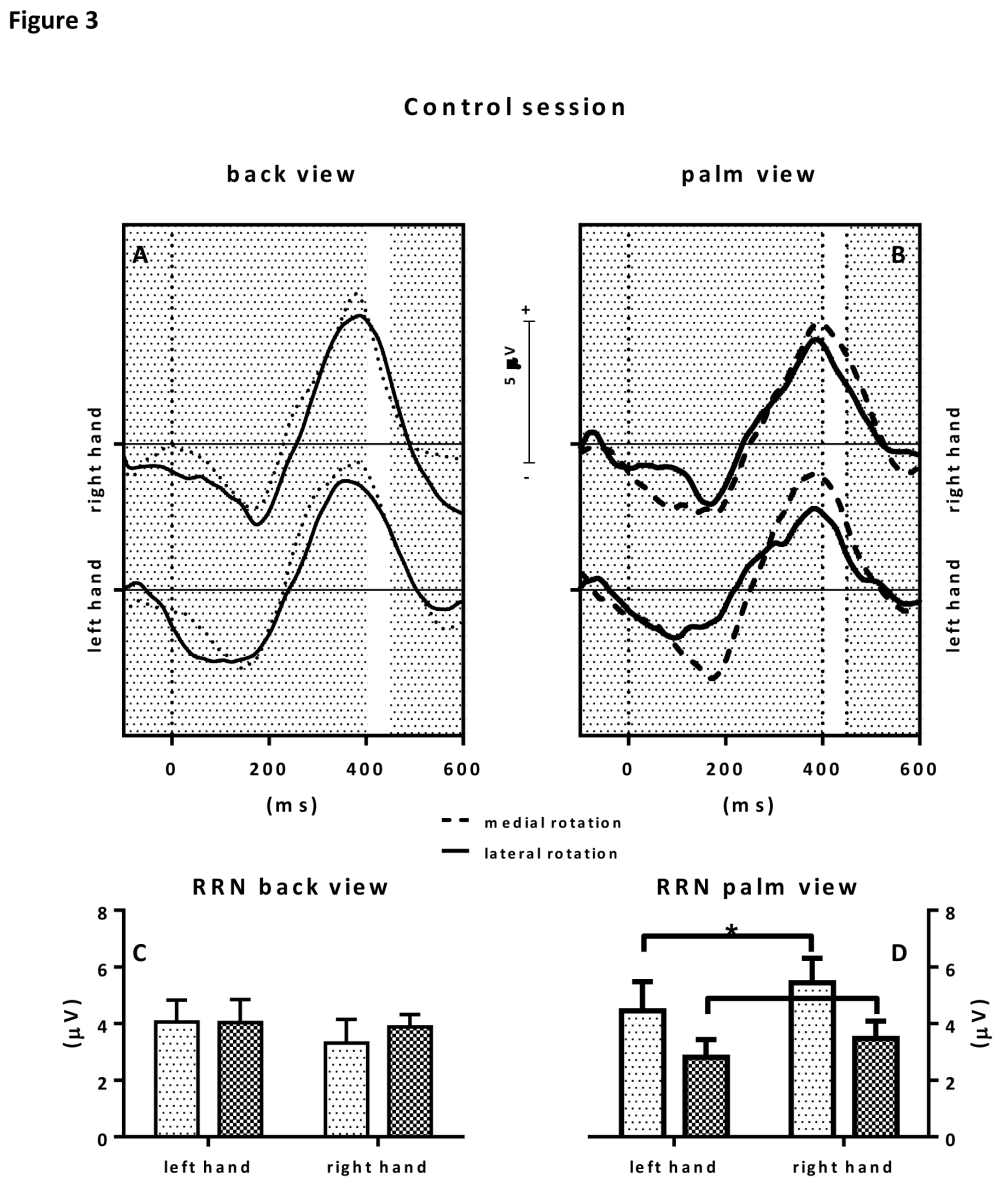
Figure 3 shows the results for the control hand posture condition. The upper panels show the grand average ERPs to back view stimuli (upper left panel) and palm view stimuli (upper right panel). Grand averages to medially rotated hand stimuli are depicted in dotted lines, grand average ERPs to laterally rotated hand stimuli are depicted in solid lines. The selected RRN window is highlighted by a white area between 400-450 ms after stimulus presentation (t=0). Consecutive RRN mean amplitude values (in µV) with error bars are depicted in the bar graphs below. On the lower left, RRNs to back view stimuli are depicted whereas on the lower right, RRNs to palm view stimuli are depicted.

In the control condition, statistical analysis of the ERP RRN amplitudes showed a significant rotation x view interaction F(1,9)=11.1; *p*=0.009; ƞ ^2^=.55. Separate analyses were conducted for back view and palm view stimuli. With respect to back view stimuli, no significant main effects for hand (left or right) or rotation (medial or lateral), nor an interaction effect was observed. With respect to palm view stimuli a main rotation effects was observed with p<.05 revealing a stronger RRN effect for laterally rotated hand stimuli than medially rotated hand stimuli. See also figure 3. IES scores showed no effects.

### Hands in inward rotated position

**Figure 4:**
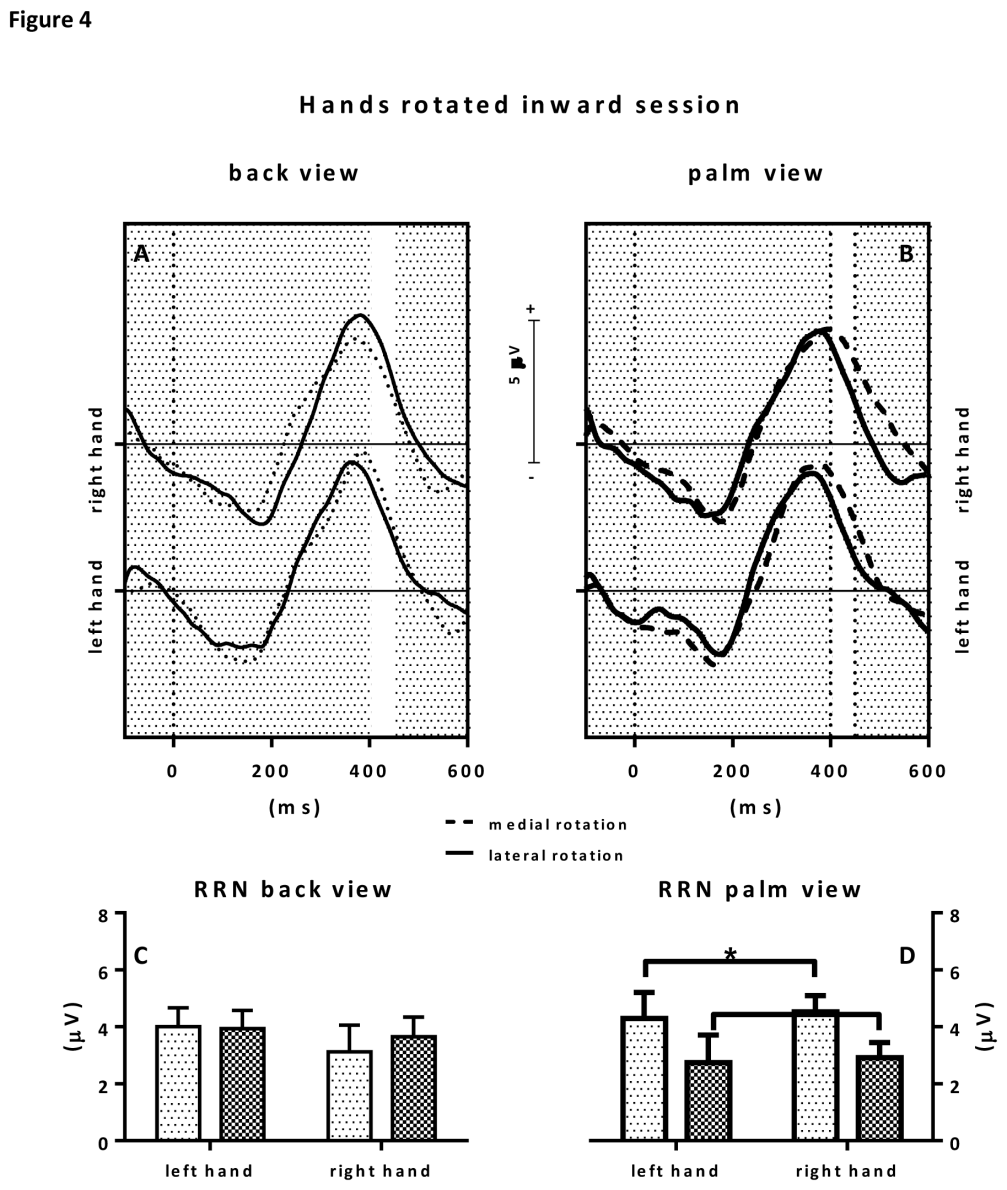
Figure 4 shows the results for the hand posture inward rotated condition. The upper panels show the grand average ERPs to back view stimuli (upper left panel) and palm view stimuli (upper right panel). Grand averages to medially rotated hand stimuli are depicted in dotted lines, grand average ERPs to laterally rotated hand stimuli are depicted in solid lines. The selected RRN window is highlighted by a white area between 400-450 ms after stimulus presentation (t=0). Consecutive RRN mean amplitude values (in µV) with error bars are depicted in the bar graphs below. On the lower left, RRNs to back view stimuli are depicted whereas on the lower right, RRNs to palm view stimuli are depicted.

Statistical analysis of the ERP RRN amplitudes showed a trend towards a rotation x view interaction F(1, 9)=4.76; *p*=0.057; ƞ ^2^=.35. No main effects were observed. Post hoc analyses were conducted for back view and palm view stimuli separately. With respect to back view stimuli, no significant main effects for hand (left or right) or rotation (medial or lateral), nor an interaction effect was observed. With respect to palm view stimuli a main rotation effects was observed with p<.05 revealing a stronger RRN effect for laterally rotated hand stimuli than medially rotated hand stimuli. See also figure 4. IES scores showed a view effect (F(1, 9)=12.67; p=0.006; ƞ^2^=.59) and a rotation x hand interaction effect (F(1, 9)=7.33; p=0.024; ƞ^2^=.449). Post-hoc tests revealed a main view and a main rotation effect for right hand stimuli (p<.05) with faster IES towards back view stimuli (M = 255.2; SD = 31.23) than palm view stimuli (M = 348.9; SD = 29.14).

### Hand outward rotated position

**Figure 5:**
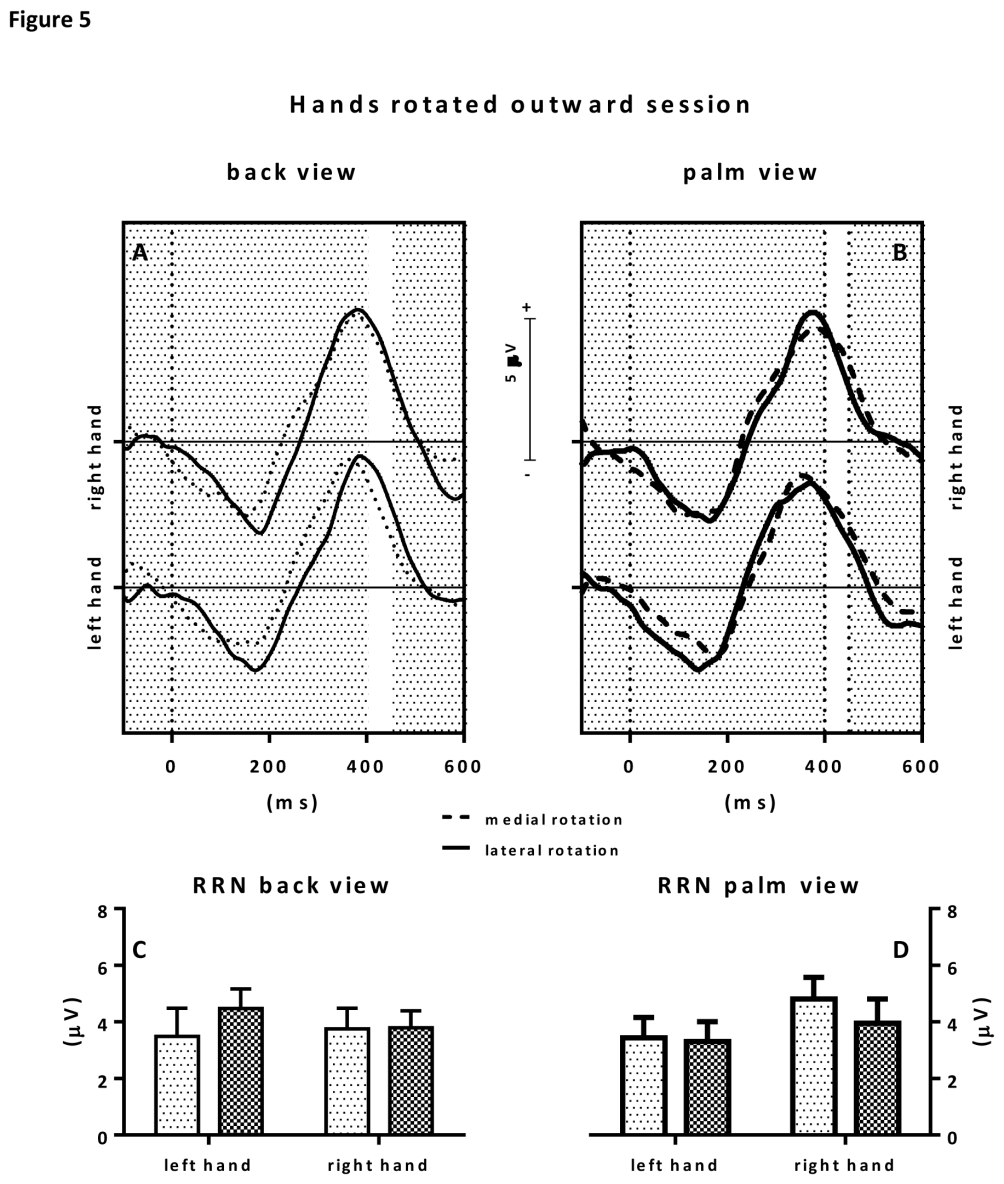
Figure 5 shows the results for the hand posture outward rotated condition. The upper panels show the grand average ERPs to back view stimuli (upper left panel) and palm view stimuli (upper right panel). Grand averages to medially rotated hand stimuli are depicted in dotted lines, grand average ERPs to laterally rotated hand stimuli are depicted in solid lines. The selected RRN window is highlighted by a white area between 400-450 ms after stimulus presentation (t=0). Consecutive RRN mean amplitude values (in µV) with error bars are depicted in the bar graphs below. On the lower left, RRNs to back view stimuli are depicted whereas on the lower right, RRNs to palm view stimuli are depicted.

Statistical analysis of the ERP RRN amplitudes showed a strong trend towards a hand x view interaction (F(1, 9)=5.07; p=0.051; ƞ^2^=.36). No main effects were observed. Although left hand stimuli seemed to elicit a more marked RRN, post-hoc tests revealed no significant effects. See also figure 5. IES scores showed a rotation x hand interaction effect (F(1, 9)=7.33; p=0.028; ƞ^2^=.43). Post-hoc tests however revealed no significant effects.

## Discussion

The main aim of the present study was to investigate the effect of hand orientation on a mental imagery task -the HLJ task- with the use of ERP measures. The current study employed a modified version of the HLJ task with three separate hand posture conditions. In each condition hands were in a different position during execution of the HLJ task. We recorded the RRN component amplitudes of the visual ERPs together with efficiency scores (IES) in a delayed response task. As expected, and in line with our previous study [16] laterally rotated palm view stimuli lead to a more marked RRN than medially rotated palm view stimuli. This study showed that this RRN effect was observed when participants had their hands positioned in either a straight (control) or an inward rotated posture but disappeared when participants had their own hand positioned in an outward rotated posture. These ERP-RRN results correspond with a 1^st^ person kinesthetic approach in solving the HLJ task.

Because we employed a delayed reaction paradigm in the present study in order to avoid motor contamination of the concurrent ERP, efficiency scores might have been obscured by a bottom effect. Normally, more efficient responses are observed for back view as compared to palm view stimuli and might be (partly) explained by visual familiarity [15,30,31]. it has been proposed that when only processing back view stimuli, participants are not engaged in MI, whereas when both palm view and back view are presented participants engage in MI, especially with respect to the palm view stimuli [32].

ERPs revealed the classically reported rotation related negativity (RRN). The RRN is an increased negativity superimposed on the descending part of the P300 between 400-450 ms after presentation of a stimulus and has been strongly linked to rotation direction in the HLJ [14,16]. As expected, a rotation effect on the RRN occurred specifically when palm view stimuli were presented. Indeed, several previous studies that employed a design with only back view stimuli, failed to find clear evidence of the involvement of MI to solve the task at hand [13,16]. With respect to the processing of back view stimuli, it has been hypothesized that we may engage in more visually based strategies such as locating the thumb in order to solve the problem [16]. When presenting palm view stimuli, however, both increased RTs for laterally rotated hands and a more marked RRN have been reported [13,14,16]. This strengthens the idea that the RRN specifically reflects activity accompanying MI.

As expected, we observed that when participants performed the HLJ task, laterally rotated palm view stimuli lead to a more marked RRN than medially rotated palm view stimuli when the participants had their hands positioned in either a straight (control) or an inward rotated position. Ter Horst et al. [16] detected a similar effect in their study of the HLJ task. The finding that laterally rotated stimuli caused a more marked RRN (in the control condition and hands inward rotated position condition) is consistent with the literature [13,14,16]. It is argued that a more effortful transformation in the lateral condition may reflect biomechanical constraints affecting MI. Ter Horst et al. [33], argued that the likelihood of engaging in MI increases relative to the number of axes of rotation. Thus, starting from a palms down position, imagining our hand in a lateral palms up position involves two axes of rotation. Previous studies have found support for this, with findings of longer reaction times and actual movement times for laterally rotated hands [10,34]. Thus, it can be argued that a more marked RRN for laterally rotated hand stimuli reflects participant’s engagement in a process of MI that is based on biomechanical representations [13]. This effect disappeared when participants had their own hand positioned in an outward rotated position, probably due to the increased congruency of hand posture and presented hand pictures. Apparently, with a congruent hand orientation (hands positioned in an outward rotated fashion) judging the otherwise more demanding laterally rotated hand stimuli seems to be facilitated, resulting in a disappearance of the RRN. This is in line with earlier observation that with increased difficulty (i.e. increased rotation angles; lateral rotations versus medial rotations; left hands versus right hands) the RRN becomes more marked. Thus, the absence of the RRN in the hands rotated outward position condition suggests a facilitation of judging pictures of laterally oriented hands when participants have their own hands placed in a congruent position.

So far, previous RRN results with the HLJ task have supported a MI approach in solving this task. That is, the RRN is modulated according to biomechanical constraints of presented palm view hand pictures, i.e. lateral rotated palm view stimuli elicit a more marked RRN since moving ones’ hand in such a position would experience enhanced biomechanical constrains than moving ones hand in a medial direction [13,14,16]. However, the main aim of the present study is how our hand position affects the RRN and whether the RRN results argue for a 1^st^ or 3^rd^ person approach of MI when solving the HLJ task. If hand posture manipulations affect the RRN within the HLJ task this would support a kinesthetic 1^st^ person approach of MI.

We observed that RRN effects disappeared in the hands positioned outward condition. This result may be interpreted in several ways. First, the lack of a rotation effect could indicate that judgments were independent of biomechanical constraints (i.e., participants did not employ MI in this condition). Possibly, the less comfortable and unnatural posture of the hands in the outward condition may have prevented participants from engaging in MI. Indeed, Sirigu and Duhamel [34,35] argued that certain postures may facilitate our engagement in MI, for instance the control condition simulates a ‘ready for action’ stance, thus priming the use of a MI strategy. It is possible that a hand orientation such as having ones hands rotated laterally may diminish such a ‘ready for action’ effect [32].

Alternatively, we propose that our result might reflect a facilitating effect of posture. That is, the difficulty of identifying laterally rotated hand stimuli (as reflected by more marked RRNs for lateral stimuli in the control and hands positioned inward conditions) may have been eliminated by a congruency between posture and stimulus in the lateral condition. Such a result supports the view that the process of MI involved in this task depends on both motor and proprioceptive mechanisms, thus arguing for a 1^st^ person kinesthetic understanding of MI in which participants rely on a ‘simulation’ of a motor movement.

## Conclusions

In conclusion, we propose that posture manipulation affect ERP RRN results thus supporting a kinesthetic MI approach in solving the HLJ task. Recently, MI has received a lot of interest with respect to its proposed application in rehabilitation after stroke and other impairments [17,36,37]. In addition, several studies have demonstrated that MI indeed provides additional benefits to conventional physical therapy [38-41]. The present study suggests that in order to be able to estimate if patients are indeed involved in MI during training sessions, ERP methodology might provide a useful tool in order to visualize the otherwise covert behavior of MI.
